# Psychiatry Residents' Views on Quality of Psychotherapy Training in Pakistan: A Cross-Sectional Survey

**DOI:** 10.1192/bjo.2024.276

**Published:** 2024-08-01

**Authors:** Aazeen Khan, Raja Adnan Ahmed

**Affiliations:** 1 King Edward Medical University, Lahore, Pakistan; 2 Swansea Bay University Health Board Ty Garngoch Hospital Hospital Road, Swansea, United Kingdom

## Abstract

**Aims:**

Several modalities of psychotherapies have an established therapeutic evidence base for many psychiatric disorders. Stakeholders around the world including the Royal College of Psychiatrists, recommend training of psychiatry trainees in psychotherapy as part of Psychiatry training. However, the quality and quantity of training in psychotherapy differ across different regions. Psychiatry training programmes in high-income countries are regularly audited to ensure minimum standards of training in psychotherapies among psychiatry trainees. There is a lack of reporting regarding psychotherapy training in low- and middle-income countries such as Pakistan. This study explores the experiences of Pakistan-based psychiatry residents regarding their psychotherapy training within the fellowship programme of the College of Physicians and Surgeons (FCPS) Pakistan.

**Methods:**

This study employs a mixed-method survey approach, targeting psychiatry trainees registered with College of Physicians and Surgeons (CPSP) four-year training programme (FCPS) across different cities of Pakistan. Utilising a convenience sampling strategy supplemented by the snowball sampling method, an electronic survey was disseminated using social media platform over a 4-week period. The survey was anonymous and structured into three sections; essential demographic data of the participants, experiences with psychotherapy training, and open-ended questions allowing participants to freely express their thoughts and insights on improving psychotherapy training in Pakistan.

**Results:**

Out of the 41 responses received, the majority were female respondents, totalling 27 (65%). All participants were FCPS trainees at various stages of their training, hailing from ten different cities across Pakistan. Findings indicated that 61% of respondents reported insufficient time to learn, understand, and apply psychotherapy techniques, while 53% identified a deficiency in supervision.

In terms of therapeutic exposure, a predominant 34 participants (82%) encountered Cognitive Behavioural Therapy during their training. Mindfulness, Dialectical Behaviour Therapy, and Family Therapy each were reported by 12 respondents (30%). Interestingly, 34 of the respondents (82%) noted an increased interest in psychotherapy since starting their psychiatric training. However, only 20 respondents (48%), felt confident in delivering psychotherapy independently.

A recurring theme emerged from the feedback: participants advocated for a more structured psychotherapy training program, emphasising the need for dedicated time specifically allocated for supervision and practical learning opportunities.

**Conclusion:**

This survey highlights that FCPS Psychiatry Residents in Pakistan are keen to learn more about psychotherapy. However, identified shortcomings in delivery, structure, and supervision suggest a need for comprehensive reforms. The findings emphasise on refining the psychotherapy training in low- and middle-income countries.

